# Tracking Cognitive Health With Wearables in Telerehabilitation Female Participants: Could Nighttime Sleep Measures Be Used as Sex-Specific Digital Endpoints?

**DOI:** 10.2196/81318

**Published:** 2026-03-05

**Authors:** Stephanie J Zawada, Louis Faust, Emma Fortune

**Affiliations:** 1 Division of Health Care Delivery Research, Mayo Clinic, 200 1st Street SW, Rochester, MN, 55905, United States, 1 480-342-5693; 2 Robert D and Patricia E Kern Center for the Science of Health Care Delivery, Mayo Clinic, Rochester, MN, United States

**Keywords:** cardiopulmonary health, cognition, dementia, digital health, sleep, wearables, women's health

## Abstract

While changes in brain structure are common across the lifespan, it is difficult to differentiate benign variations from early disease pathogenesis, especially in patients participating in home-based rehabilitation. Cognitive decline is frequently linked with normative aging, but its early detection can facilitate preventative interventions, particularly in patients at high risk of cognitive impairment and dementia, such as older women. Although women have fewer modifiable risk factors for dementia than men, wearables have the potential to establish new digital endpoints that facilitate the management and/or prevention of abnormal brain aging. Sleep, which is necessary for maintaining overall brain health, is one behavior tracked via wearables, for which an ever-growing body of pilot and validation phase studies exists, yet endpoints defining optimal sleep are not one-size-fits-all, as individual chronotype variability necessitates new strategies to personalize care. Though sex-based differences in circadian rhythm are well established, little is understood about which sleep measures and thresholds are uniquely important to cognitive health in women, particularly those with high comorbid burden. In this viewpoint, we discuss recent findings on the use of wearables to track sleep and cognitive health in women, while highlighting challenges and opportunities for health outcomes and clinical trial researchers seeking to implement meaningful digital endpoints in future telerehabilitation programs.

## Introduction

With the rapid adoption of wearables in home-based rehabilitation, also known as telerehabilitation, an emerging need exists to establish clinically meaningful digital endpoints, “precisely defined variable[s] intended to reflect an outcome of interest that is statistically analyzed to address a particular research question” [1]. For telerehabilitation participants with neurologic or functional deficits, such as those with recent hospitalizations for stroke, chronic obstructive pulmonary disease, or coronary artery disease, research has shown the effectiveness of interventions to monitor and track exercise through digital endpoints derived from wearable devices, such as steps per day [2-4]. Beyond physical activity monitoring, the application of wearables to track cognitive health, defined as the “improvement, maintenance, or minimal decline of cognitive function and absence, delay of onset, or slowing the progression of dementia,” is on the rise, yet it remains understudied in telerehabilitation [5-8].

The monitoring of cognitive health during telerehabilitation has typically relied on videoconferencing, requiring clinical assessments of participant status; however, the link between cognitive health and sleep, which can be passively observed using wearables, is well documented [9,10]. Historically, research investigating the sleep-cognition relationship has primarily featured univariate correlations between scores obtained from cognitive assessments and self-reported sleep duration [11]. Both short and long sleep durations, outside the optimal window (7‐9 h per night), have been associated with cognitive decline; however, the application of wearable devices has introduced new inconsistencies regarding this association [12]. Though self-report bias remains a known confounder in analyses relying on survey instruments, arbitrary thresholds, such as device-specific cutoffs for nighttime versus daytime sleep, used in sleep data processing might contribute to conflicting results regarding wearable-derived sleep duration and cognitive health [13]. The generalizability of these results to telerehabilitation populations remains unknown [14].

Despite limitations, wearable devices offer an enhanced approach to elucidating the sleep-cognition link by capturing objective measures beyond sleep duration, from sleep fragmentation scores to nighttime blood oxygen saturation, offering a never-before-seen multidimensional view of sleep [13]. To realize the potential of multidimensional sleep data, their use in high-acuity populations, such as patients undergoing rehabilitation, requires the validation and specific consideration of known risk factors for cognitive decline, such as age and cardiovascular disease (CVD) [15]. With the goal of 1 day operationalizing sleep measures as digital endpoints reflective of cognitive health, we encourage researchers to take a thoughtful approach to processing sleep data, particularly in female populations, as hormonal shifts across the lifespan contribute to evolving sleep chronotypes. Considering that women have fewer modifiable risk factors for dementia than men but are at a higher risk of cognitive impairment, new strategies to promote cognitive health in women are urgently needed [16,17]. The application of sleep monitoring during telerehabilitation could be one approach to unlock new discoveries in women’s cognitive health—if meaningful digital endpoints can be created.

Such insights would be exceptionally helpful for clinicians overseeing telerehabilitation cohorts, wherein subtle cognitive deficits might slowly emerge and not be accurately captured in surveys due to participant self-report bias or cognitive decline impacting self-management [18]. Rather than providing a comprehensive review of wearables in the telerehabilitation space in this viewpoint, we aim to emphasize the aspects of a few recent wearables studies involving sleep measures beyond sleep duration alone that may be translated into clinical trials and outcomes research, preparing investigators to formulate exploratory sleep digital endpoints related to cognition and women’s health in telerehabilitation populations.

## Cognitive Performance and Sleep Onset and Regularity in Women

One recent study by Swanson et al [19] examined relationships between cognitive performance and sleep measures in older adult women who enrolled in the Study of Women’s Health Across the Nation. In the subsample of participants who completed the at-home wearables study during a follow-up visit in 2015‐2016 (n=1177; mean age=65 y), participants were instructed to wear an Actiwatch-2 (Philips Respironics) on their nondominant wrist for 7 days to record critical sleep measures: timing (average midpoint from sleep onset to wake) and regularity (midpoint SD) [20]. Since the Actiwatch-2 uses accelerometer sensors to record movement, its sleep onset and regularity measures are derived from periods of nonmovement during nighttime [21]. Cognitive performance was measured via motor, visual, learning, and memory questions in these validated assessments: the East Boston Memory Test, Symbol Digit Modalities Test, and Digit Span Backwards exam.

Metabolic burden in participants was high, with 63.5% being hypertensive and 17.6% being diabetic. The majority of participants had 2 or more comorbidities (54.9%) and a waist circumference indicating central obesity (59.9%). Importantly, at baseline, the average participant was at intermediate risk for a heart attack or stroke within a decade (mean atherosclerotic cardiovascular disease risk score (ASCVD)=9%). The authors adjusted linear regression models for relevant covariates to explore associations between real-world sleep measures and cognitive performance, reporting *β* coefficients representing the change in cognitive measure for a 1-unit change in sleep measure. Irregular sleep timing was linked with improved working memory (*β*=.50; *P*=.004) and worse delayed (*β*=−.36; *P*=.006) and immediate verbal memory (*β*=−.29*; P*=.02). While late sleep timing, defined as a midpoint after 4:00 AM, was associated with decreased processing speed (*β*=−1.80; *P*=.008), early sleep timing (a midpoint before 2:00 AM) was associated with worse delayed verbal memory (*β*=−.37*; P*=.047). Notably, a sensitivity analysis revealed that the magnitude of the effect of sleep irregularity on working memory was greater in participants with hypertension (interaction *β*=−3.35, *P*=.04). In addition, as the magnitude of ASCVD risk increased, so did the strength of the association between early sleep timing and delayed verbal memory (interaction *β*=−8.83; *P*=.03), drawing attention to the impact of CVD risk factors on sleep and cognitive decline in older women.

These findings highlight the potential for wearable-derived sleep measures to elucidate cognitive health; however, they specifically focused on older adult women. Hormones influence sleep characteristics, particularly in women, and their associations, as well as their directionalities and magnitudes, may not generalize well to younger cohorts. These results support existing literature that finds irregular sleep associated with gray matter atrophy and increased CVD risk via high β-amyloid burden, both potential contributors to cognitive decline [22].

Only ASCVD and hypertension revealed significant interactions with sleep-cognition associations in Swanson et al’s [19] study, both of which are linked with increasing white matter hyperintensity volume in middle age [23,24]. It is plausible that the lack of associations for some factors, such as diabetes, which is a known risk factor for dementia, represents an age-related baseline for otherwise “healthy” women. The circadian clock controls blood pressure and the hypothalamic-pituitary-adrenal axis, which regulates blood glucose levels. As age and dementia risk increase, the circadian clock weakens [25]. After hypertension emerges, vascular changes influence the development of white matter hyperintensities, amyloid-β deposits, and cerebrovascular disease–related atrophy, all contributing to cognitive decline [26]. Downstream, these changes, hypothalamic-pituitary-adrenal dysfunction, may, in some cases, lead to diabetes or central obesity via insulin resistance, which then modulates the association between some sleep measures and cognition [27,28]. Therefore, digital endpoints related to sleep might require adjusting for comorbid conditions beyond CVD, but such discussion is outside the scope of this viewpoint.

## Sex-Specific Cardiovascular Disease Risk and Sleep Patterns

One method to further elucidate the link between sleep and cognitive health in women during telerehabilitation could involve the thoughtful consideration of CVD risk when designing sleep digital endpoints.

Expanding on the CVD results presented by Swanson et al [19], the results from Nikbakhtian et al [29] highlight the importance of considering sex-specific CVD markers when tracking sleep remotely. They investigated the role of sleep onset in CVD development using a large population cohort study, the UK Biobank (n=103,712; 57.9% female; aged 43‐79 y), which captured 1 week of real-world sleep data via the wearable Axivity AX3 accelerometer (Open Lab, Newcastle University).

In contrast to male participants, who mostly experienced sleep onset later in the night, women usually experienced sleep onset between 10:00 and 11:00 PM, the sleep onset time associated with the lowest incidence of CVD. Adjusting for relevant risk factors, sleep regularity, and sleep duration, Cox proportional hazards models generated hazard ratios (HRs), summarizing the risk of CVD diagnosis occurring in a cohort stratified by sleep onset time. Here, the associations between sleep onset time and CVD risk persisted, yielding Cox proportional hazards ratios (HRs) of 1.25 (CI 1.02‐1.52; *P*=.03), 1.24 (CI 1.10‐1.39; *P*<.005), and 1.12 (CI 1.01‐1.25; *P*=.04) for sleep onset times of >12:00 AM, <10:00 PM, and 11:00-11:59 PM, respectively. In sex-specific models, the associations between sleep onset times of <10:00 PM (HR=1.63; CI 1.20‐2.21; *P*<.005) and >12:00 AM (HR=1.63; CI 1.20‐2.21; *P*<.005) were more pronounced in female participants. In contrast, only the association between sleep onset time of <10:00 PM and CVD was significant in male participants.

CVD and Alzheimer disease, the most common form of dementia, often share markers of systemic pathogenesis, as amyloid-β deposits can accumulate in the heart muscle, vessels, and brain as the diseases progress [30]. Moreover, hypertension contributes to brain structural changes implicated in cognitive decline, a finding more pronounced in menopausal and post-menopausal women, partly due to hormonal changes [31]. Thus, particular attention should be paid to sex-specific CVD risk when developing sleep digital endpoints for cognitive health, as CVD risk factors might help predict cognitive decline.

In addition to plausible mechanisms by which CVD contributes to cognitive decline, systematic reviews have reinforced the need for the proactive management of cognitive health in patients with CVD [32]. For example, Eggermont et al [32] concluded that interventions stimulating cognitive function should be routinely considered when developing treatment protocols for patients with CVD. Specific to telerehabilitation, for which a limited number of studies on CVD and cognition are published (n=9), Dabbaghipour et al’s [33] systematic review outlined the need for rehabilitation programs to track cognitive health with the goal of improving treatment adherence and quality of life in participants. Combined, these reviews highlight the need for cognitive health monitoring to consider CVD risk factors, especially hypertension, though neither review assessed the role of sleep. One potential way to operationalize CVD risk in the development of sleep digital endpoints might be to stratify endpoints based on hypertension severity, with patients experiencing worse hypertension in need of aggressive sleep interventions [31,34-37].

## Cognitive Impairment and Pulmonary Function During Sleep

The links between pulmonary markers and cardiovascular as well as cognitive health are well documented [32,38]. Not only is low blood oxygen saturation associated with a high risk of cognitive impairment, but it is also implicated in CVD exacerbations, forcing changes in cardiovascular and brain structures that affect sleep patterns and quality [39,40]. One commonly studied pulmonary marker is peripheral capillary oxygen saturation (SpO_2_). Readily recorded by photoplethysmography (PPG) sensors in many wearable devices, SpO_2_ serves as an indirect measure of lung diffusion, quantifying the concentration of oxygen molecules transported in blood from inhalation [41].

Extending the results of Swanson et al’s [19] work, Ding et al [42] used the SleepImage Ring to track SpO_2_ during sleep. In this study of older adult participants (n=62; 67.7% women; mean age=74 y; 80.5% cognitively intact), intraclass correlation coefficients (ICCs), which measure agreement across multiple timepoints, were reported for measures recorded over a 3-day sleep monitoring period, with ICCs equal to or greater than 0.70 indicating reliability. Using SleepImage Ring data from real-world settings, mean SpO_2_ during sleep (ICC range: 0.75‐0.77) was stable over 3 nights in participants without cognitive impairment; however, this finding did not persist in participants with cognitive impairment (ICC range=0.68‐0.83). In addition to sleep onset and regularity measures, mean SpO_2_ during sleep may inform digital endpoints that help researchers differentiate between individuals with and without cognitive impairment.

The findings from another exploratory study conducted in clinical settings, with a middle-aged cohort of obstructive sleep apnea (OSA) patients (n=207; 44.4% female; mean age=49), extend the link in Ding et al’s [42] study between pulmonary function during sleep and cognitive health [43]. Thorisdottir et al [43] used data from the Embla PSG device (Flaga) obtained over a 1-night observational period and scores obtained from the Rey Auditory Verbal Learning Test. Reporting *β* coefficients, they found that mean SpO_2_ during sleep was significantly associated with both immediate recall (*β*=−.171; *P*<.022) and total recall (*β*=−.188; *P*<.007).

Considering the results of these studies and prior work demonstrating that verbal memory is impacted by blood oxygen saturation, it is plausible that nighttime SpO_2_ might specifically facilitate the indirect observation of verbal memory [44-46]. Though the external validity of Thorisdottir et al’s [43] findings beyond patients with OSA is unknown, the underdiagnosis of OSA in the general population is high, with estimates of 40% to 80% of patients with CVD also experiencing OSA [47,48]. As such, the results of sleep study analyses that exclude patients with an OSA diagnosis may inadvertently include those with undiagnosed OSA. Due to the limited number of large population studies with comparable wearable devices, a thoughtful approach to assessing the current sleep-cognition literature available, on populations with and without OSA, is vital.

Ding et al’s [42] study focused on older adults, while that of Thorisdottir et al [43] involved a middle-aged cohort. As such, the interpretation of SpO_2_ measures might necessitate stratification by age, especially considering that Thorisdottir et al’s [43] study was conducted in clinical settings and may not generalize to the real world. The findings by Tam et al [49] using the SleepImage Ring offer support for age-stratification of pulmonary measures from wearables when formulating endpoints. Tam et al [49] hypothesized that sleep quality in women decreases after menopause. They conducted the first 1-night study with composite variables derived from built-in accelerometer sensors to capture movement in addition to SpO_2_ from the PPG sensor.

Tam et al [49] performed subgroup analyses with one-way ANOVA, comparing the means of different measures across age-stratified cohorts to detect a statistically significant difference. Tam et al [49] used the SleepImage Ring and proprietary measures derived directly from the ring’s data stream. In this cohort (n=1444; 48.8% female; mean age=54), Tam et al [49] observed a drop in the Sleep Quality Index, a composite measure using both PPG and accelerometer sensor data to score sleep fragmentation, sleep quality, sleep stability, and sleep periodicity, in women 51 years of age and older. After the age of 50, women also experienced a greater increase than men in the apnea-hypopnea index, which uses SpO_2_ to quantify shallow and paused breathing episodes, as well as the Arousal Index, which relies on accelerometer data to quantify shifts from deep sleep to near wakefulness. Though the authors did not investigate cognitive health in participants across stratified age groups, future researchers could address this gap to better understand if composite sleep measures offer new insights beyond unidimensional measures, such as sleep duration, into cognitive changes across the lifespan [49]. Furthermore, an increase in the Arousal index is associated with a higher risk of CVD, suggesting that an age of 50 years might be an appropriate threshold for researchers to explore for adjusting sleep digital endpoints for CVD risk factors [50].

## Multidimensional View of Cognitive Health and Sleep

The potential for these wearable-derived endpoints, should they prove to be more clinically meaningful than conventional self-reported sleep endpoints, to provide more accurate analyses can advance the design of remote monitoring programs in telerehabilitation and beyond. They can also inform the development of more effective sleep interventions targeting populations with high comorbid burden, such as telerehabilitation patients.

To date, the only peer-reviewed multivariate analysis of sleep and cognition necessitated a multi-step statistical analysis. Qin et al [51] used the Oura Ring (Oura) for 14‐28 days to capture 23 sleep measures and investigate associations between sleep measures and cognitive domains in adults who participated in the Singapore Chinese Health Study. A total of 7 cognitive domains (verbal memory, attention, visuospatial ability, visual memory, language, executive function, and processing speed) were assessed via the following validated instruments: Rey Auditory Verbal Learning Test, Boston Naming Test, Associative Learning Test, Brief Visuospatial Memory Test-Revised, Color Trail Tests 1 and 2, Design Fluency Test, WAIS-III, and Symbol Digit Modality Test. First, scores were standardized from each test to T scores (mean 50, SD 10). Then, for cognitive domains with more than 1 test score, the reported score was summarized as the average of all test scores for that domain.

After adjusting for confounding variables, including age and sex, Qin et al [51] applied a series of statistical techniques to identify significant associations between sleep and cognitive health. First, they used partial least squares correlation (PLSC), a statistical technique using 2 matrices—in this case, a matrix of multiple sleep measures and a matrix of multiple cognition scores—to highlight optimal relationships between sleep, as a multidimensional concept, and cognition, also as a multidimensional concept. Their analysis of data obtained from adult Chinese participants over the age of 65 years (n=773; 51.1% female; mean age=75; 1.2% with cognitive impairment) found that the first PLSC component contributed the majority of covariance (82%) between cognition and sleep scores (*r=*0.2; *P*<.001).

After generating the covariance matrix, they applied singular value decomposition to generate latent variables representing the maximum covariance between sleep and cognitive measures. For each latent variable with a significant correlation value (Pearson’s *r*), 5000 bootstrap tests were run to identify measures contributing to the first PLSC component. They identified robust contributions to the first PLSC component for 11 sleep measures and 3 cognitive domains. Qin et al [51] found 8 sleep regularity measures (Sleep Regularity Index [SRI], Sleep Fragmentation Index [SFI] Intraindividual Standard Deviation [iSD], Wake-After-Sleep-Onset (WASO) iSD, Efficiency iSD, Total Sleep Time iSD, Time in Bed iSD, Wake Time iSD, Bedtime iSD) and 3 continuity measures (Efficiency, WASO, SFI) contributed to the sleep-cognition relationship. All but SRI (which tracked whether an individual was awake or asleep at the same time each day), SFI (which quantified nighttime movement and sleep epochs lasting less than 1 min), and SFI iSD were extracted directly from the Oura dataset. Executive function, verbal memory, and processing speed were identified as relevant cognitive domains, each of which is associated with age-related decline [52].

Finally, to probe for associations between specific sleep measures and cognitive domains, a partial correlation analysis was performed, identifying only weak correlation coefficients between sleep measures and cognitive domains. Among sleep regularity measures, a higher SRI was associated with better processing speed (*r*=0.17) and improved executive function (*r*=0.17). Additionally, as sleep efficiency (efficiency iSD) was less regular, processing speed declined (*r*=0.15). In contrast, among sleep continuity measures, only high sleep efficiency was associated with better processing speed (*r*=0.11).

Using multidimensional, objective sleep measures, Qin et al’s [51] results represent a significant step forward in elucidating the relationship between sleep and cognition; however, their analyses did not include sleep measures derived from relevant biomarkers, like blood pressure or pulmonary function, highlighting a gap in the literature for future researchers to fill.

## Challenges and Recommendations

Despite the use cases for sleep digital endpoints in telerehabilitation highlighted above, no systematic reviews or meta-analyses have been published on this topic. The disparate study methods, device types, and sleep measures used contribute to the lack of evidence synthesis on the topic. As such, the statistical significance, magnitude, and directionality of the findings presented in this viewpoint should be interpreted as exploratory and not absolute, with future research required to use these measures in practice and real-world settings. Fundamentally, to develop useful sleep digital endpoints, robust and reliable associations between sleep measures and cognition must be established.

This viewpoint highlights multiple trends in the sleep-cognition space to achieve this goal. First, more large cohort studies using wearables that collect multidimensional data from across relevant biomarkers—including sleep biomarkers like SpO_2_ in addition to sleep timing measures—must be conducted. Longitudinal sleep data captured beyond 1-week periods should be prioritized [53-55]. Accurate longitudinal monitoring is especially relevant when conducting aging research in cognitive impairment and dementia, when prevalence rates change drastically over short epochs. For instance, dementia prevalence is <1% in adults under the age of 65 years [56]. After the age of 65 years, that number shifts to between 3% and 11% and climbs to over 30% in adults over 85 years of age [56]. When reviewing studies with less than 1 week of data, the results should be interpreted cautiously, as the limited samples may not be representative of an individual’s sleep routine. Enrolling larger cohorts for longer periods will facilitate the development of standardized protocols for processing sleep wearable data and, subsequently, validating digital endpoints and related thresholds in telerehabilitation populations [57].

Second, across the recent studies presented in this piece, measures of sleep regularity were the most frequently associated with cognitive health. Therefore, we encourage researchers to prioritize studying sleep regularity across the lifespan. Considering that 2 individuals could sleep for the same duration during the night while experiencing separate disturbances, sleep regularity measures quantify individual sleep habits across days more sufficiently than duration measures alone.

Finally, the findings outlined in this viewpoint offer an exploratory rationale for the development of sleep digital endpoints stratified by age. With stark changes in pulmonary function and cardiovascular risk linked to sleep patterns in menopausal women, age might serve as a proxy for changes in hormone concentration. Alternatively, the inclusion of blood biomarker data to stratify sleep digital endpoints might offer more precise monitoring in telerehabilitation programs. Regardless, establishing thresholds relying on sleep data to bifurcate healthy versus impaired cognition necessitates a closer look by researchers. These issues are complicated by the fact that individual sleep chronotypes change across the lifespan, necessitating robust methods that account for within-person variability, rather than relying exclusively on population-based thresholds [58]. Such an approach is appropriate for dynamic monitoring in real-world settings, automatically accounting for variability in the environment.

Our viewpoint outlines multiple opportunities for researchers to advance knowledge in the pursuit of developing sleep digital endpoints in telerehabilitation programs. Beyond the lack of systematic reviews and meta-analyses assessing sleep measures relevant to telerehabilitation, no research into cognitive health and SpO_2_ measures during sleep stratified by age has been published. Additionally, no multivariate analyses assessing sleep measures and cognitive measures have included nighttime biomarker measures, like SpO_2_ or blood pressure levels. Of note, for studies exclusively focused on older adults, the associations between sleep measures and cognitive domains might be subject to survival bias and require additional investigation to confirm results across age cohorts. Addressing these gaps will create a starting point for more robust research into the sleep-cognition relationship, advancing the promise of wearables.

These studies may have implications for the future of sleep monitoring research in detecting cognitive decline in women. First, the influence of cardiovascular and pulmonary risk factors on the association between sleep and cognition underscores the importance of considering comorbid burden when interpreting cognitive health data [59]. Next, these remote monitoring approaches can reach women in rural areas who might otherwise be unable to participate in in-clinic monitoring, thereby addressing barriers and adherence issues by introducing enhanced flexibility in rehabilitation care [60].

Beyond the potential of monitoring research on sleep and cognition, numerous real-world barriers limit the implementation of sleep digital endpoints, even when they are validated in research. Historically, most validation research on wearable device measures was performed using research-grade devices. In recent years, with the growing acceptance of consumer-grade devices, the shift to validating consumer-grade devices has introduced new complexities. For instance, consumer-grade wearables often generate proprietary composite measures with limited documentation. Also, the interpretation of results derived from wearable devices requires special attention paid to the device version, as software and hardware updates might introduce unknown variability in the data collected [61].

Another barrier to real-world implementation of sleep digital endpoints is devising feasible strategies to integrate wearable data streams into clinical and research workflows. Should researchers pinpoint meaningful sleep measures and thresholds for digital endpoints tracking cognitive health in women, the data collection, wrangling, and analysis processes are resource intensive. If a feasible and cybersecure data pipeline infrastructure for wearables is designed and provides actionable information to clinical and research teams, the demand for evidence-based interventions to help participants will increase [62,63]. For telerehabilitation participants, this might require interventions that can be delivered outside of clinical settings, from home health visits and/or digital tools. Recently, novel applications of digitally delivered cognitive behavioral therapy have shown promise with regard to improving sleep measures in patients. Cognitive behavioral therapy interventions might confer a protective effect on cognitive health during telerehabilitation, one day being measured by digital endpoints [64-66]. Virtual reality–based support tools and virtual companions could also be offered in tandem with conventional telerehabilitation programs [67-69]. Notably, unclear reimbursement and payment pathways for remote monitoring of sleep, particularly in the United States, remain a key bottleneck to its widespread adoption [70].

The application of artificial intelligence (AI), such as machine learning methods, to swiftly detect trends in patient sleep and cognition can augment workflows and make the implementation of digital endpoints a reality. For sleep digital endpoint implementations that rely on AI to review 24/7 data from patients, threshold levels will likely evolve, as more data are fed into databases on which AI is trained [71]. This requires additional review and analysis of evolving AI tools. In addition to the workflow barriers, identifying patients who want to participate in sleep monitoring, as well as determining how to troubleshoot tech problems remotely, can be challenging. At the time of this publication, wearable monitoring research over longitudinal periods appears promising in older adults, even in those with cognitive impairment and dementia [28,72].

Multiple study design limitations complicate the interpretation of sleep data from wearables. First, the studies surveyed in this viewpoint reflect significant heterogeneity with regard to methodologies and primary endpoints [73-75]. Therefore, deriving actionable information for women’s health from objective sleep data remains elusive. Also, the proportion of participants in sleep studies with wearables has been overwhelmingly male (61%) [76]. Next, the location of wear for wearables, such as wrist versus ring finger, is sometimes limited during rehabilitation due to functional deficits and can influence results. Since the body of literature in this space is continually growing, there is a need for reviews summarizing and comparing the results by location of wear, particularly in female participants.

Summarizing the recent studies outlined in this viewpoint, we recommend that researchers consider the sleep measures and cognitive variables in Table 1 as a starting point to design digital endpoints from measures obtained via wearable sensors. Researchers should also consider the impact of age and CVD risk factors, including hypertension, as they develop clinically meaningful thresholds specific to female patients. Theoretically, a rudimentary sleep digital endpoint for cognitive health in female patients between the ages of 50 and 60 years old without hypertension might establish a target sleep onset between 10 PM and 12 AM with stable mean SpO_2_ during sleep 7 days per week. This outcome of interest incorporates multidimensional sleep measures (sleep onset and SpO_2_), adjusted for hypertensive status and age, setting an optimal threshold for monitoring cognitive health indirectly. To be operationalized in clinical settings, values that fall outside the aforementioned thresholds might trigger clinician review. While plausible based on the findings reviewed in this viewpoint, this digital endpoint is purely hypothetical. Thus, research beyond what is currently available is required to formulate sleep digital endpoints in light of comorbid burden.

The list of measures in Table 1 is by no means exhaustive, with new digital measures ripe for application to tracking cognitive health emerging every year. For example, 1 exploratory sleep measure to investigate across the lifespan is nighttime movement by age. In an illustrative example using an arm-worn sensor (Everion) to track sleeping habits in patients with multiple sclerosis, Moebus et al [77] found that age correlated strongly with movement during the night, a measure unstudied in the context of female cognitive health and telerehabilitation. Other novel measures include sound-based measures of sleep, using a smartphone-compatible microphone, and applying AI algorithms to classify sleep stages in real-world settings [78].

**Table 1.** Sleep measures and related cognitive variables outlined in recent research.

Wearable-derived sleep measure	Cognitive variable (instrument)
Sleep onset (time) and regularity (standard deviation)	Working memory (Digit Span Backward) Verbal memory (East Boston Memory Test) Processing speed (Symbol Digit Modalities Test)
SpO_2_^a^ (mean, min, max)	Cognitive impairment (Montreal Cognitive Assessment) Rey auditory verbal learning
Sleep Regularity Index (Oura-specific)	Processing speed (Colour Trail Test 1; Symbol Digit Modality Test)
Sleep efficiency	Executive function (Colour Trail Test 2; Design Fluency Test)

a SpO_2_: oxygen saturation.

Overall, these studies build upon current literature by highlighting potentially modifiable sleep measures, such as sleep onset, sleep regularity, sleep continuity, or SpO_2_ during sleep, that can inform the development of digital endpoints for cognitive health. The age- and sex-specific nature of the results as well as CVD considerations outlined provide an early-stage rationale for developing more personalized digital endpoints for sleep stratified by known risk factors for cognitive decline [37].

## Conclusions

Historically, telerehabilitation programs have relied on physical activity endpoints to monitor and evaluate participants in real-world settings; however, wearable-derived sleep measures might augment conventional telerehabilitation protocols by providing insights into cognitive health, addressing a critical gap for older female participants who are at higher risk of cognitive decline. Ultimately, the studies outlined above offer an early-stage rationale for investigating emerging sleep measures as multidimensional endpoints for cognitive health in female patients. We recommend that the contributions of sleep and nighttime pulmonary measures to cognitive decline in women be explored further using wearables and adjusting for cardiovascular factors across middle and late adulthood. As no systematic reviews or meta-analyses have been published on this topic, the specific findings presented in this viewpoint should be interpreted circumspectly. Nonetheless, the combined findings demonstrate how researchers might hypothetically apply real-world sleep data from wearables to develop clinically meaningful digital endpoints of cognitive health for women, while being mindful of adjustments necessary to account for comorbid burden. By addressing the gaps in knowledge outlined in this viewpoint, researchers can make strides in transforming sleep measures into digital endpoints for cognitive health interventions, enabling proactive care at home to delay or stop the onset of cognitive decline in women during critical periods, such as rehabilitation.

## Abbreviations

AI: artificial intelligence
ASCVD: atherosclerotic cardiovascular
CVD: cardiovascular disease
HR: hazard ratio
ICC: intraclass correlation coefficient
iSD: intraindividual standard deviation
OSA: obstructive sleep apnea
PLSC: partial least squares correlation
PPG: photoplethysmography
SFI: sleep fragmentation index
SpO_2_: oxygen saturation
SRI: sleep regularity index
